# Pre-Treatment Computed Tomography Radiomics for Predicting the Response to Neoadjuvant Chemoradiation in Locally Advanced Rectal Cancer: A Retrospective Study

**DOI:** 10.3389/fonc.2022.850774

**Published:** 2022-05-10

**Authors:** Yitao Mao, Qian Pei, Yan Fu, Haipeng Liu, Changyong Chen, Haiping Li, Guanghui Gong, Hongling Yin, Peipei Pang, Huashan Lin, Biaoxiang Xu, Hongyan Zai, Xiaoping Yi, Bihong T. Chen

**Affiliations:** ^1^Department of Radiology, Xiangya Hospital, Central South University, Changsha, China; ^2^National Clinical Research Center for Geriatric Disorders (Xiangya Hospital), Central South University, Changsha, China; ^3^Department of General Surgery, Xiangya Hospital, Central South University, Changsha, China; ^4^Hunan Key Laboratory of Skin Cancer and Psoriasis, Xiangya Hospital, Central South University, Changsha, China; ^5^Department of Pathology, Xiangya Hospital, Central South University, Changsha, China; ^6^Department of Pharmaceuticals Diagnosis, General Electrics Healthcare, Changsha, China; ^7^Hunan Engineering Research Center of Skin Health and Disease, Xiangya Hospital, Central South University, Changsha, China; ^8^Department of Diagnostic Radiology, City of Hope National Medical Center, Duarte, CA, United States

**Keywords:** nomogram, spiral computed tomography, neoadjuvant therapy, rectal neoplasms, chemoradiation

## Abstract

**Background and Purpose:**

Computerized tomography (CT) scans are commonly performed to assist in diagnosis and treatment of locally advanced rectal cancer (LARC). This study assessed the usefulness of pretreatment CT-based radiomics for predicting pathological complete response (pCR) of LARC to neoadjuvant chemoradiotherapy (nCRT).

**Materials and Methods:**

Patients with LARC who underwent nCRT followed by total mesorectal excision surgery from July 2010 to December 2018 were enrolled in this retrospective study. A total of 340 radiomic features were extracted from pretreatment contrast-enhanced CT images. The most relevant features to pCR were selected using the least absolute shrinkage and selection operator (LASSO) method and a radiomic signature was generated. Predictive models were built with radiomic features and clinico-pathological variables. Model performance was assessed with decision curve analysis and was validated in an independent cohort.

**Results:**

The pCR was achieved in 44 of the 216 consecutive patients (20.4%) in this study. The model with the best performance used both radiomics and clinical variables including radiomic signatures, distance to anal verge, lymphocyte-to-monocyte ratio, and carcinoembryonic antigen. This combined model discriminated between patients with and without pCR with an area under the curve of 0.926 and 0.872 in the training and the validation cohorts, respectively. The combined model also showed better performance than models built with radiomic or clinical variables alone.

**Conclusion:**

Our combined predictive model was robust in differentiating patients with and without response to nCRT.

## Introduction

Locally advanced rectal cancer (LARC) is usually treated with neoadjuvant chemoradiotherapy (nCRT), followed by total mesorectal resection. Response to nCRT largely determines the prognosis and survival of patients with LARC (1). Patients may achieve pathological complete response (pCR), which is defined as the complete absence of tumor cells in the resected specimen, while some patients may only have partial response or no response. The reported pCR rate of LACR to nCRT is relatively low, ranging from 10% to 30% (1–4). Patients who achieve a pCR may adopt a conservative “watch and wait” strategy without surgery (5). Therefore, the extent of response to nCRT affects clinical decision-making and determines whether patients should be directed to aggressive surgical treatment. Currently, the gold standard for pCR relies on pathological confirmation of a surgical specimen. There is a need for developing non-invasive methods to reliably predict the response to nCRT and to avoid surgery for patients with pCR.

Predictors of pCR include clinical demographics, tumor morphology, blood cell counts, serum oncological indicators, protein expression, gene profiles, conventional radiological imaging features, the time interval between nCRT and surgical resection. However, these predictors have produced inconsistent results with some indicating their usefulness in predicting pCR and some indicating otherwise (2, 6–8). In addition to CT imaging, current efforts and studies with other imaging modalities have been performed to assist in prediction of pathological responses with promising results. PET/CT has the advantage of detecting the metabolically active rectal cancer and metastases for staging; and semiquantitative parameters derived from sequential PET/CT imaging may be used to predict response (9, 10). Endorectal ultrasound (EUS) affords direct visualization and access for biopsy of rectal cancer and its adjacent lymphadenopathy, which has also been used to predict response to nCRT (11). MRI may provide exquisite anatomical details on tumor morphology and its association with adjacent structure (12, 13). However, these additional imaging modalities are more time-consuming and costly as compared to CT imaging. In addition, some patients may not be able to tolerate the endorectal probe for EUS or the endorectal coil for rectal MRI. As CT remains to be the most commonly used imaging modality in clinical practice, it is prudent to assess its potential for predicting pCR in patients with LARC.

Radiomics detects tumor image features through computational analysis, which may reflect biological properties of tumors. It has been used to predict treatment response in patients with gastrointestinal tumors including rectal cancer (14). Prior studies have shown robust performance of predictive models for pathological response, achieving the area under the curve of 0.98 in models combining radiomic signature and clinical parameters such as tumor length (15). Radiomic features reflecting tumor heterogeneity such as entropy, skewness and kurtosis have been indicated as most relevant for predicting response to treatment (14). However, few radiomic studies have focused on these relevant radiomic features to determine tumor heterogeneity for predicting pCR in patients with LARC (2, 8, 16). In addition, prior radiomics studies of rectal cancer had small sample sizes without external validation, predisposing to overfitting and issues of generalizability (17). More work is needed to develop non-invasive radiomic approaches for cancer diagnosis and treatment.

Pretreatment contrast-enhanced CT for radiotherapy treatment planning is routinely acquired for patients with LARC prior to nCRT, which has provided a platform for predictive modeling of clinical response through imaging analysis. However, there have been conflicting results using radiomics to predict response to treatment, with some studies indicating its usefulness and some studies indicating otherwise (2, 8, 18–21). In the present study, we analyzed pretreatment contrast-enhanced CT images acquired for radiotherapy treatment planning, extracted radiomic features, and incorporated clinicopathological risk factors to build models for predicting pCR in patients with LARC undergoing nCRT. We hypothesized that the combined prediction model built with both radiomics and clinicopathological parameters could be useful for differentiating patients with pCR from patients without pCR.

## Materials and Methods

### Patients

**Figure 1:**
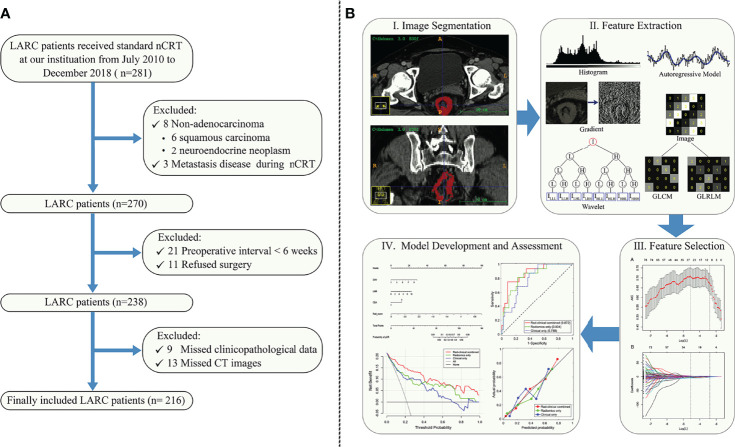
**(A)** Study enrollment flow chart of patients with locally advanced rectal cancer (LARC) who received neoadjuvant chemoradiotherapy (nCRT). **(B)** Workflow for the method section. (I) Tumor segmentation on the CT images. (II) Radiomic feature extraction. Six classes of radiomic features were extracted from the tumor, including histogram, gradient, gray level co-occurrence matrix (GLCM), gray level run-length matrix (GLRLM), autoregressive model, and wavelet texture. (III) Radiomic feature selection. (IV) Predictive modelling and nomogram.

Consecutive adult patients with LARC who underwent nCRT followed by total mesorectal excision with or without a pathological confirmation of pCR from July 2010 to December 2018 were retrospectively enrolled into this study and their medical information was extracted from our institutional database. The patients were randomly allocated to the training or validation cohort at a ratio of 7:3 using computer-generated random numbers. Details of the patient recruiting process as well as the exclusion criteria are presented in **Figure 1A**.

The nCRT was carried out according to standard protocols. Briefly, nCRT was delivered to the whole pelvis at a dose of 46-50 Gy in 23-25 fractions (2 Gy/fraction, 5 days/week) with concurrent oral capecitabine. During the interval between nCRT and surgery, additional chemotherapy was administrated with the regimens consisting of mainly capecitabine plus oxaliplatin, called CapeOX, or a combination of leucovorin calcium, fluorouracil, and oxaliplatin called FOLFOX6.

The following 20 pretreatment clinicopathological variables were collected from medical records: gender, age, overall CT value (i.e., the mean value of CT density in Hounsfield Unit in the region-of-interest of the tumor), distance to the anal verge (DAV), pathological grade, hemoglobin (HGB) level, platelet counts, neutrophil-to-lymphocyte ratio (NLR), lymphocyte-to-monocyte ratio (LMR), platelet-to-lymphocyte ratio (PLR), albumin concentration, globin concentration, albumin-to–globulin ratio (A/G), cholesterol level, high-density lipoprotein (HDL), low-density lipoprotein (LDL), occult blood (OB), carcinoembryonic antigen (CEA), carbohydrate antigen 199 (CA199), and carbohydrate antigen 125 (CA125). This retrospective study was approved by the Ethics Committee and Institutional Review Board in our institution, and written informed consent was waived due to the retrospective nature of this study.

### Pathological Re-Assessment

The surgical specimens, which were embedded in paraffin and sliced into 4-mm-thick sections, were re-assessed by two experienced gastrointestinal pathologists (GG and HY, with 6 and 25 years of experience, respectively) to evaluate pCR according to the established criteria (22, 23). Briefly, a pCR was defined as no viable tumor cells present in the bowel wall (T stage) or regional nodes (N stage) at T0N0 (complete regression). Changes in TNM staging were also included in the assessment of tumor response.

### CT Imaging Acquisition

All patients underwent a routine contrast-enhanced CT scan for radiotherapy treatment planning in supine position on a 16- multi-detector row spiral computed tomography (Brilliance 16, Philipps) scanner. The contrast-enhanced CT images were performed after intravenous injection of 90-100 ml of iodinated contrast material (Ultravist 370, Bayer Schering Pharma, Berlin, Germany) at a rate of 3.0-3.5 ml/second. Enhanced images at portal venous-phase (scanned with fixed delay time of 60-70 seconds) were obtained for all patients. The CT images were retrieved from the Picture Archiving and Communication System (PACS, Carestream, Canada) and transferred to an external workstation (Leonardo; Siemens Medical Solutions, Forchheim, Germany). All CT images were reconstructed with a thickness of 3 mm. Normalization with 256 bins was performed on all original CT images using the gray-scale discretization method before extracting the radiomic features (Analysis Kit software, version V3.0.0.R, GE Healthcare).

### CT Imaging Analysis

CT images for each patient were reviewed independently by two abdominal radiologists (reader 1: YM and reader 2: HL, with 6 and 25 years of experience, respectively). The radiologists were blinded to all information about the patients including the radiological and clinicopathological data. Using the conformal region-of-interest approach, CT attenuation values in Hounsfield units were measured at the largest dimension of the tumor on axial images. Regions of interest were placed in three locations within the tumor and the average CT attenuation values were calculated. In case of discrepancy in the opinions of the two radiologists, a third senior radiologist (CC, with 33 years of experience) would be involved in assessment and consensus would be reached through discussion.

### Texture Feature Extraction

For each patient, a representative axial image with the largest cross-sectional area of the tumor (thickness: 3 mm) was selected by the two abdominal radiologists (reader 1 and reader 2), who made the decision together. Digital Imaging and Communications in Medicine (DICOM) Works software (version 1.3.5) and MaZda Version 4.6 (Institute of Electronics, Technical University of Lodz, Poland) were used for transfer and texture analysis, respectively. All tumor contouring was reviewed and validated by two senior abdominal radiologists (Haiping Li and CC, both with more than 25 years of experience in interpreting abdominal CT images).

For each patient, 340 quantitative texture features were automatically generated using the MaZda software from each region-of-interest file, including a gray-level histogram, a gradient, a run-length matrix, a co-occurrence matrix, an autoregressive model and a wavelet transform analysis.

### Reproducibility of Texture Feature Extraction

Reproducibility of texture feature extraction was analyzed by two radiologists performing independent segmentations of the CT images. The inter-observer (reader 1 versus reader 2) and intra-observer (reader 1 twice within a two-week period) correlation coefficient (ICC) values were evaluated on the 50 randomly chosen images. The final consistency was evaluated by the following criteria applied to the ICC value: <0.20 for poor reproducibility, 0.21-0.40 for fair reproducibility, 0.40–0.60 for moderate reproducibility, 0.61–0.80 for good reproducibility and 0.81–1.00 for excellent reproducibility. Generally, an ICC greater than 0.75 was regarded as indication of good agreement. Reader 1 completed the workflow for the remaining images.

### Radiomic Feature Selection

According to the criterion of ICC > 0.75, the features with an ICC value less than or equal to 0.75 were excluded and were not included for further analyses. To build the radiomic signature (Rad-score), the least absolute shrinkage and selection operator (LASSO) method was used to select the most relevant features (24). A Rad-score was calculated for each patient using a linear combination of those selected features, with their respective coefficients weighted in the combination. The Rad-score was deemed as an independent variable along with the other clinicopathological variables. Univariate and multivariate logistic regression was used to select the independent predictors for pCR, with the *p* value set at 0.05.

### Model Assessment

The discrimination performance of the models was calculated using the area under the curve (AUC) of the receiver operating characteristic (ROC) curves. Calibration curves, which evaluated accuracy of a predictive model, were created by plotting the observed probabilities against the model-predicted probabilities *via* a bootstrap method (resampling iteration = 1000). The Hosmer-Lemeshow test was performed to test if the calibration curve was significantly different from the ideal curve (25). Decision curve analysis for evaluating clinical usefulness of a model was implemented by quantifying the net benefits for a range of threshold probabilities in the validation dataset (26, 27). Additionally, the predictive values of the Rad-score based on CT features for T and N status (T0 *vs.* non-T0, N0 *vs.* non-N0) were also measured in terms of AUC.

We used feature selection and logistic regression for our statistical modelling method in this study. A prior study by Huang et al. used logistic regression to develop a predictive model for lymph node metastasis in colorectal cancer and achieved robust performance (28). Logistic regression could be used to deal with biomedical datasets which usually have unbalanced outcome variables. In addition, because the outcomes of logistic regression were probabilities, this method has made it possible for subsequent calibration analysis, nomogram plotting, and decision curve analysis for comprehensive performance assessment of a predictive model.

### Statistical Analysis

All statistical analyses were conducted with R software version 3.6.1 (http://www.Rproject.org) using the following packages: “glmnet”, “rms”, “pROC”, “rmda”, “ggplot2”, and “broom”. The nomogram was created using “rms”. The AUCs for different models were compared using the deLong test (29). All statistical tests were two-sided, with statistical significance set at 0.05. The workflow for this study is presented in **Figure 1B**.

Quality assessment of this study was performed according to the radiomics quality score (RQS) which has provided standardized criteria and reporting guidelines to minimize bias and enhance prediction models in radiomic research (30). Most points lost in our study were due to it being a retrospective study. For instance, we could not add the 7 points designated for prospective validation of a radiomics signature in an appropriate trial. The total RQS for this study was 22 points out of a maximal score of 36 points, which was reasonably good for a radiomic study. A systematic review of radiomic studies predicting response to treatment in gastrointestinal cancers showed the RQS ranging from −4 to 23 points, with a median of 5 points for the 60 studies included in the review (14). A detailed assessment of the 16 key components for RQS was presented in the Supplementary files (**Table S1**).

## Results

### Patient Characteristics

A total of 216 patients with LARC were included in our study. The overall pCR rate was 20.37% (44/216). Clinicopathological characteristics of patients in the training cohort (n = 151) and the validation cohort (n=65) are summarized in **Table 1**. The training and validation cohorts were similar in terms of the pCR rate (19.88% and 21.54%, respectively, *p* = 0.912), as well as the other clinicopathological variables (all *p* > 0.05).

**Table 1:** Patient characteristics.

	Training cohort (n = 151)	Validation cohort (n = 65)	*p* value
**Gender**			0.981
** Male**	91	40	
** Female**	60	25	
**Age (years)**	53 (46-60)	54 (46-62)	0.835
**Overall CT density value (HU)**	55.9 (50.4-62.0)	57.1 (50.8-64.1)	0.762
**Distance to anal verge (cm)**	5.0 (4.0-6.0)	5.0 (4.0-7.0)	0.432
**Pathological grade**			0.662
** Well/moderately differentiated**	99	42	
**Poorly differentiated**	43	21	
**Mucinous carcinoma**	9	2	
**Hemoglobin (g/L)**	132 (120-145)	135 (122-145)	0.312
**Platelet counts (×10^9^/L)**	235 (192-297)	240 (206-276)	0.481
**Neutrophil to lymphocyte ratio**	2.3 (1.8-3.1)	2.2 (1.8-2.9)	0.894
**Lymphocyte to monocyte ratio**	3.4 (3.0-4.5)	4.3 (3.2-5.3)	0.088
**Platelet to lymphocyte ratio**	151.2 (111.4-196.9)	138.1 (110.0-182.5)	0.311
**Albumin (g/L)**	43.2 (40.1-45.6)	43.1 (39.7-45.7)	0.883
**Globin (g/L)**	27.8 (24.9-30.9)	27.9 (24.1-29.8)	0.531
**Albumin/globin (A/G)**	1.6 (1.4-1.7)	1.6 (1.4-1.8)	0.437
**Cholesterol (mmol/L)**	4.8 (4.0-5.4)	4.8 (4.3-5.6)	0.497
**High density lipoprotein (mmol/L)**	1.2 (1.0-1.5)	1.3 (1.1-1.4)	0.949
**Low density lipoprotein (mmol/L)**	2.9 (2.3-3.4)	2.9 (2.5-3.6)	0.280
**Occult blood**			0.803
** Positive**	124	55	
** Negative**	27	10	
**Carcinoembryonic antigen (ng/mL)**			0.911
** < 5**	87	44	
** ≥ 5**	55	30	
**Carbohydrate antigen 199 (kU/L)**			0.991
** Normal (< 35)**	133	58	
** Abnormal (≥ 35)**	18	7	
**Carbohydrate antigen 125 (kU/L)**			0.737
** Normal (< 35)**	144	61	
** Abnormal (≥ 35)**	7	4	
**Pathological complete response**			0.924
** Positive**	30	14	
** Negative**	121	51	
**T status after nCRT**			0.304
** T0**	30	17	
** Non-T0**	121	48	
**N status after nCRT**			0.469
** N0**	109	50	
** Non-N0**	42	15	

Data are either n or median (lower-upper quartile) unless otherwise indicated. Comparison between the two cohorts uses either two sample Student t-test/Mann–Whitney U test for normally/non-normally distributed continuous variables and χ2 test for categorical variables. **CT,** computed tomography. **HU,** Hounsfield units.

### Radiomic Feature Selection and Radiomic Signature

A total of 340 radiomic features were extracted from the CT images for each patient. Initially, 65 low-stability (ICC ≤ 0.75) features were excluded, and the remaining 275 features were included in the final analysis. Using LASSO logistic regression on the training cohort, 264 additional features were excluded because of their coefficients being squeezed to zero based on the one standard error of the minimum criterion (**Supplementary Files: Figure S1**). The remaining 11 features were linearly added for calculation of the Rad-score (see **Supplementary Files: Figure S2**) and weighted with their respective non-zero coefficients (28).

### Predictors for Building Models

The results of univariate and multivariate logistic regression are summarized in **Table 2**. The final predictors were the following: distance to the anal verge, lymphocyte-to-monocyte ratio, CEA, and Rad-score. Three predictive models were built. Model 1 was built with all four final predictors; Model 2 was built with Rad-score alone; and Model 3 was built with the three remaining clinical predictors after excluding the Rad-score.

**Table 2:** Binary logistic regression analysis of risk factors for pathological complete response.

Factors	Univariate analysis			Multivariate analysis		
	OR	95% CI	*p* value	OR	95% CI	*p* value
**Gender**	0.989	0.427-2.292	0.980			
**Age**	0.980	0.560-1.717	0.945			
**Overall CT density value (HU)**	1.259	0.867-1.826	0.226			
**Distance to anal verge**	1.822	1.228-2.703	0.003	2.236	1.267-3.944	0.006
**Pathological grade**	3.027	0.656-13.961	0.156			
**Hemoglobin**	1.190	0.727-1.948	0.489			
**Platelet counts**	0.979	0.584-1.642	0.936			
**Neutrophil to lymphocyte ratio**	1.265	0.896-1.691	0.461			
**Lymphocyte to monocyte ratio**	2.895	1.733-4.834	<0.001	2.241	1.075-4.672	0.031
**Platelet to lymphocyte ratio**	0.967	0.591-1.581	0.894			
**Albumin**	1.115	0.647-1.921	0.696			
**Globin**	0.706	0.399-1.248	0.231			
**Albumin/globin (A/G)**	1.302	0.866-1.959	0.205			
**Cholesterol**	1.242	0.702-2.196	0.456			
**High density lipoprotein**	0.834	0.477-1.459	0.525			
**Low density lipoprotein**	1.252	0.665-2.356	0.486			
**Occult blood**	0.583	0.219-1.553	0.280			
**Carcinoembryonic antigen**	0.221	0.084-0.582	0.002	0.169	0.042-0.683	0.013
**Carbohydrate antigen 199**	0.841	0.606-1.168	0.302			
**Carbohydrate antigen 125**	0.873	0.624-1.221	0.427			
**Rad-score**	10.580	3.815-31.302	< 0.001	20.581	5.396-78.502	<0.001

CI, confidence interval; CT, computed tomography; HU, Hounsfield Units; OR, odds ratio.

### Model Performance

**Figure 2:**
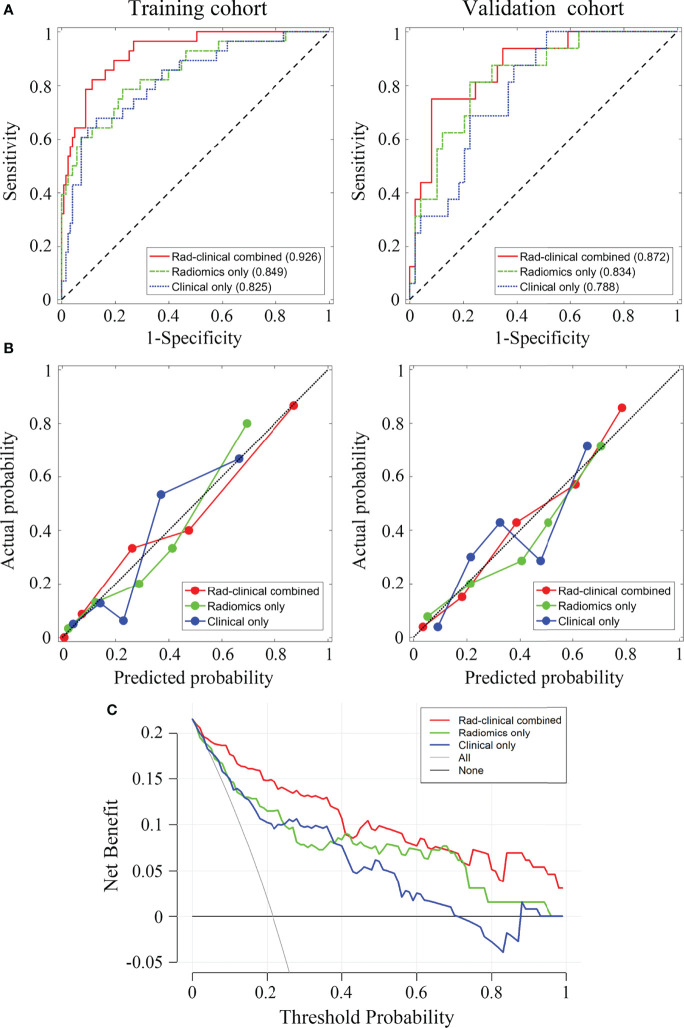
The receiver operating characteristic curves for the three prediction models and the corresponding decision curves. **(A)** The receiver operating characteristic (ROC) curves for training and validation cohort, the area under the curve of each model is displayed in parentheses. **(B)** Calibration curves for training and validation cohorts. **(C)** Decision curves for the three models. Red, combined radiomic and clinical data model; green, radiomic model; blue, clinical data model.

The ROC analyses for the three models are presented in **Figure 2A**. The AUCs of the ROC curves for the training cohort were 0.926 (95% CI: 0.878-0.974), 0.849 (95% CI: 0.765-0.933), and 0.825 (95% CI: 0.738-0.913) in Model 1, Model 2, and Model 3, respectively. The DeLong test showed that the AUC of Model 1 was significantly higher than the other two (both *p* < 0.05), while Model 2 and Model 3 had similar AUC values (*p* > 0.05). For the validation cohort, the AUCs were 0.872 (95% CI: 0.777-0.968), 0.834 (95% CI: 0.726-0.942), and 0.788 (95% CI: 0.676-0.900) for Models 1, 2, and 3, respectively. No significant AUC differences were found among any two of the three models for the validation cohort (all *p* > 0.05). The discrimination performance of the three models is presented in **Table 3**. The AUC cut-off values in **Table 3** were determined based on Youden index maximization criterion.

**Table 3:** Performance of the three predictive models.

Metrics	Model 1 (combining radiomics and clinical data)		Model 2 (radiomics only)		Model 3 (clinical data only)	
	Training	Validation	Training	Validation	Training	Validation
**AUC**	0.926	0.872	0.849	0.834	0.825	0.788
**Accuracy**	0.868	0.862	0.768	0.769	0.828	0.600
**Sensitivity**	0.821	0.750	0.786	0.812	0.679	1
**Specificity**	0.886	0.918	0.772	0.776	0.870	0.490
**PPV**	0.605	0.706	0.431	0.520	0.528	0.381
**NPV**	0.956	0.917	0.940	0.925	0.922	1

AUC, area under the receiver operating characteristic curve; PPV, positive predictive value; NPV, negative predictive value.

The AUC cut-off was determined based on Youden index maximization criterion. Specifically, Youden index = true positive rate (sensitivity) – false positive rate (1-specificity). In the ROC curve, a series of Youden indices was calculated, then the maximum Youden index of this series was picked out and the corresponding value of the test variable which matched this maximum Youden index was the cut-off value.

The AUCs of Rad-score for predicting T status were 0.826 (95% CI: 0.738-0.913) and 0.811 (95% CI: 0.697-0.926) for the training and validation cohorts, respectively; The AUCs of Rad-score for predicting N status were 0.749 (95% CI: 0.654-0.844) and 0.716 (95% CI: 0.568-0.863) for the training and validation cohorts, respectively.

The calibration curves for the three models showed good consistency between the predicted pCR rate and the observed pCR rate for both the training and validation cohort (**Figure 2B**). These findings did not reach statistical significance by the Hosmer-Lemeshow test for any of the six calibration lines (3 models×2 cohorts, all *p* > 0.05), indicating good agreements with the ideal diagonal line. The calibration lines for Model 1 were visibly closer to the diagonal line, implying the best predictive accuracy.

**Figure 3:**
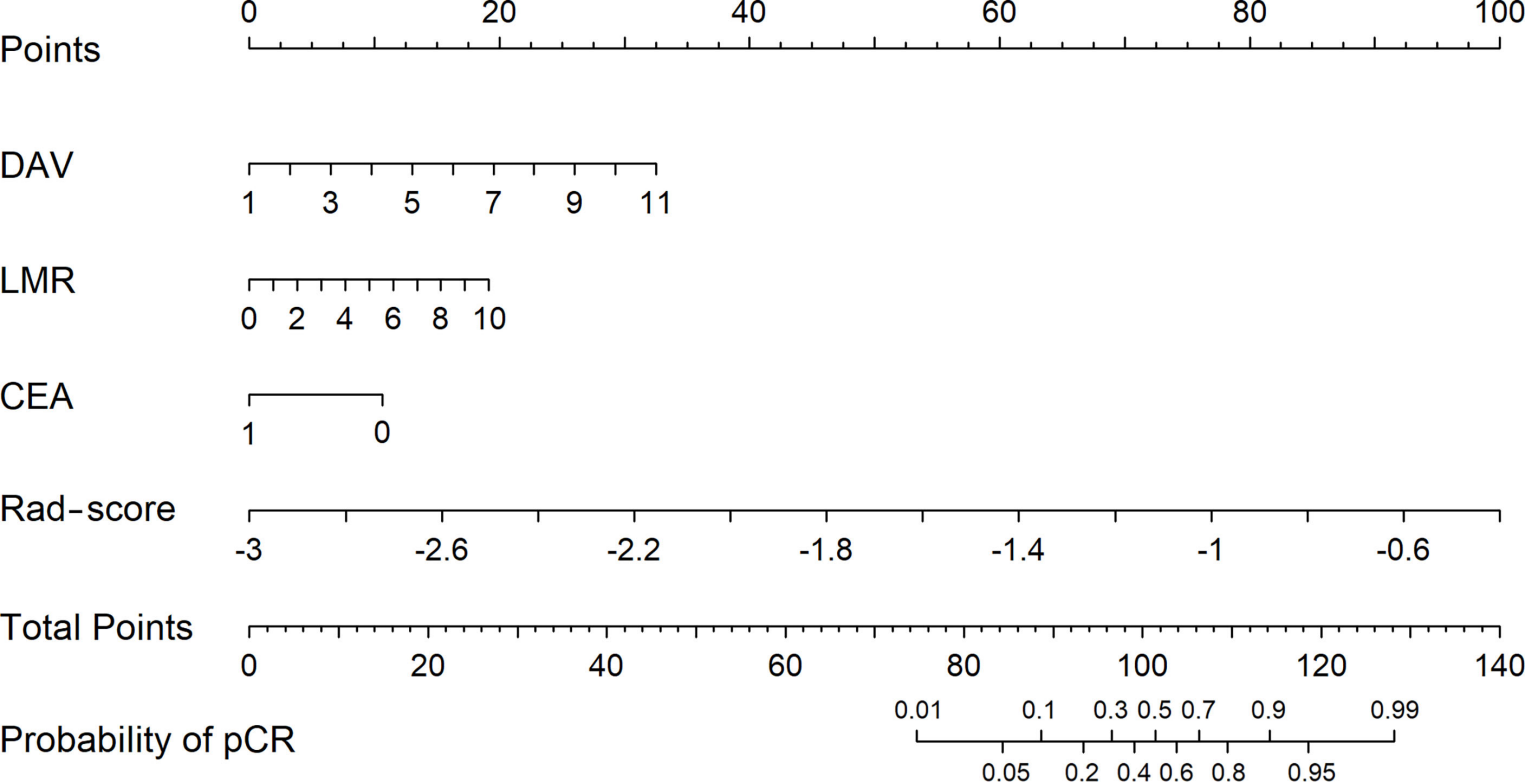
The nomogram for the model combining radiomics and clinical data.

**Figure 2C** shows that using decision curve analysis, Model 1 appears to have the widest threshold probability as well as the highest position, indicating the most clinical advantage. Nonetheless, using any of these models could be beneficial clinically. Overall, our results indicate that Model 1 is the most robust model tested in the present study. **Figure 3** shows the nomogram for Model 1.

## Discussion

In this study, we performed radiomic analysis on pretreatment radiotherapy planning CT images performed for routine clinical care and developed a radiomic nomogram to predict the response to nCRT in patients with LARC. Our predictive model, built with a combination of radiomics and clinicopathological risk factors, had reasonable performance in differentiating patients with and without pCR. The clinical usefulness of the model was also confirmed by the decision curve analysis. Our study results support the notion of using routine radiation treatment planning CT imaging to predict treatment response in clinical practice.

There is limited literature on the use of CT-based radiomics to predict the response to nCRT in patients with LARC. A prior study using a deep neural network method based on contrast-enhanced CT images achieved moderate accuracy of 80% for predicting pCR in patients with LARC receiving nCRT (16). In addition, neural network methods are difficult to understand intuitively and are considered “black box” methods for their lack of interpretability. On the other hand, our model was built with radiomic features derived directly from the tumor images, making it more relatable to clinical practice. Moreover, the prior study using deep neural network methods had a sample size of 95 subjects (16) and our model had the advantage of training and validating on a larger cohort of 216 subjects. Larger sample sizes are critical for model building because of the need to avoid overfitting of the prediction models.

Our study used contrast-enhanced CT images for radiomic analysis, which should be superior to using non-contrast enhanced images because contrast enhancement should reveal more details on the heterogenous internal architecture of malignant tumors. Two prior studies used a non-contrast-enhanced CT radiomic model to predict pCR in rectal cancer and their results were conflicting (2, 8). The model reported by Yuan et al. (2) had 83.9% accuracy and promising predictive power, while the model reported by Hamerla et al. (8) showed no predictive power for treatment response. Our results support the use of radiomics based on contrast-enhanced CT images in predicting treatment response of LARC.

CT has been the most commonly used imaging modality for diagnosis and treatment planning of various diseases. For instance, a recent study presented the feasibility of using CT images imported into a radiation treatment planning system for volumetric assessment of COVID-19 pneumonia lesions and for localizing the pulmonary lesions as a target for 3D conformal radiation therapy (31). This innovative approach is encouraging and may have potential for treatment planning of rectal cancer since nCRT also requires identification of the tumor location and volumetric assessment for radiation therapy. More work needs to be done to assess this innovative method in diagnosis and treatment of rectal cancer.

Our model combining the Rad-score with clinical features achieved robust performance. These results support further investigation of the Rad-score as a biomarker to predict the response of LARC to nCRT. Radiomic features might reflect the biological nature of tumor cells and have been found to be associated with tumor prognosis (32, 33). For instance, higher heterogeneity may indicate a more aggressive tumor with a worse prognosis while lower heterogeneity might indicate more angiogenesis with a better prognosis (33). In our study, two entropy-related features (WavEnLH_s_3, WavEnLH_s_4, see Supplemental file), indicating higher heterogeneity, were among the most relevant radiomic features filtered by the LASSO method. Both had negative coefficients, implying higher values of these features being associated with reduced likelihood of pCR. Our results were consistent with a model wherein higher entropy reflecting higher heterogeneity and worse prognosis.

The mechanism underlying the relationship between the location of rectal cancer and response to treatment is not clear yet. Our study included tumor location aiming to assess this relationship further. Prior studies have suggested that distal location of rectal cancer tended to have better prognosis, either in terms of pCR rate or in distant metastasis-free survival (7, 34–37). One study found that tumors located higher *vs.* lower in the rectum had pCR rates of 23.2% *vs.* 7.3%, respectively (34). Another study reported a similar finding, with pCR rates of 20% and 3%, for tumors higher and lower in the rectum, respectively (37). However, one study found a higher pCR rate in lower tumor location in the rectum (38). Our results were consistent with the majority of published literature showing a higher pCR rate in the tumors located higher in the rectum. We speculate that the differential blood supply, the anatomic structure, and the density of the surrounding tissue might affect the chemotherapeutic drug concentration and radiation dose in the target rectal area, resulting in different responses to treatment.

Serum inflammatory cytological biomarkers as well as tumor markers were widely studied in rectal cancer (39–41). Our model included an inflammatory cytological biomarker (i.e., LMR) and a tumor marker (i.e., CEA). In a study by Li et al. (40), LMR was found to be the most relevant factor for pCR in rectal cancer, with an AUC of 0.913. Diakos and colleagues (42) proposed that systemic inflammation may affect the tumor response through the complex interplay between local immune responses and systemic inflammation. Our results support the role of LMR in assessing treatment response of LARC. CEA is a known marker of tumor response and prognosis in gastrointestinal cancer. CEA is abnormally expressed in various malignant tumors, and higher expression has been linked to worse prognosis in colorectal cancer (43). Thus, our findings that CEA was negatively correlated to pCR were consistent with the literature. However, there were some differences in CEA cut-off determinations among the studies. For example, the cut-off value to indicate CEA-positive status was set at 5 ng/mL in some studies (44, 45), and at 2 ng/mL in other studies (46). The pCR has been linked to either the post-treatment CEA level (46, 47), the pretreatment CEA level (45, 48), or both the pre- and post-treatment CEA levels (44). Nonetheless, the potential usefulness of CEA as a prognostic biomarker for rectal cancer has been recognized and our results add to the evidence that the pretreatment CEA level may help to predict treatment response.

The prognostic factors for non-metastatic rectal cancer patients have been noted to include age, nutritional condition, tumor stage, tumor differentiation, and surgery, which may independently affect overall survival (49). Our best performing model for predicting response to nCRT incorporated both radiomics and clinical variables, such as radiomic features, distance to anal verge, lymphocyte-to-monocyte ratio (LMR), and carcinoembryonic antigen (CEA). It should not be surprising for this model to perform well as radiomic features may reflect heterogeneity of tumor cells and have been found to be associated with tumor prognosis. The location of tumor (distance to anal verge), and serological markers such as LMR and CEA are associated with biological behavior of the tumor, which may affect tumor stage and prognosis. Other prognostic factors are related to the patients’ general well-being such as frailty and nutritional status. For instance, the loss of skeletal muscle mass during nCRT has been shown to be related to lower survival for patients with rectal cancer (50). Higher BMI tends to have better survival for patients with colorectal cancer (51). More research should be performed focusing on the potentially modifiable factors such as muscle mass and body weight to enhance response to nCRT.

There were several limitations to this study. First, this was a retrospective study with uncontrolled confounding variables, such as clinical staging, nodal status and time interval between treatment initiation to surgery, making case selection bias possible and affecting the performance of our predictive model. Second, this retrospective study included a modest sample size of 216 patients from a single institution over a long enrollment period, with only 44 patients achieving pCR. The modest sample size in our study might have increased the risk of overfitting the models, and a single institution data may limit the model’s generalizability. Therefore, external validation is mandatory and should be performed in future studies. Third, differences in imaging protocols may hinder the general applicability of our results. For instance, we performed contrast-enhanced CT for radiotherapy treatment planning while patients were in supine position and other centers may use non-contrast enhanced CT imaging while their patients may assume either supine or prone position. In addition, contrast-enhanced CT may differ from facility to facility depending on the experience and expertise of the clinical practice. Furthermore, a combination of limited sample size and lack of radiomics on the CT scans acquired post-nCRT but before surgery may have missed some useful features for model building to predict response to treatment. Therefore, future studies should adopt a standardized imaging protocol among all participating institutions and should have sufficient statistical power to tease out the effect of imaging protocol on model performance. Lastly, several important variables such as gene profiles, and membrane protein biomarkers were not assessed in our study, which could have helped in assessing the potential for personalized treatment and their effects on the model performance. Future prospective multicenter studies with a large sample size are needed to validate our results and assess the clinical applications of our predictive model.

## Conclusions

This study demonstrates the usefulness of pretreatment CT radiomics for predicting the response to nCRT in patients with LARC. Our data supported the notion of using non-invasive imaging-focused approaches to assess treatment response and to guide personalized treatment for patients with rectal cancer.

## Data Availability Statement

The original contributions presented in the study are included in the article/**Supplementary Material**. Further inquiries can be directed to the corresponding authors.

## Ethics Statement

The studies involving human participants were reviewed and approved by the Ethic Committee and Institutional Review Board in Xiangya Hospital of Central South University, P. R. China (IRB No.201610070). Written informed consent for participation was not required for this study in accordance with the national legislation and the institutional requirements.

## Author Contributions

YM and XY: conceptualization, methodology development, and original draft preparation. QP, HY, and HLi: data analysis. CC, YF, HLiu, GG, and HY: resources collection. PP and HLin: scanning guidance and data collection. BX and HZ: draft revising and fund providing. BC: methodology supervision and draft revising. All authors contributed to the article and approved the submitted version.

## Funding

This study was funded in part by the National Natural Science Foundation of China (81701847), the Natural Science Foundation of Hunan Province, P.R. China (2017JJ3497, 2018JJ2641), the Xiangya-Peking University Wei Ming Clinical and Rehabilitation Research Fund (No. xywm2015I35), and China Post-doctoral Science Foundation (2018M632997).

## Conflict of Interest

Author PP and HL were employed by the company GE Healthcare.

The remaining authors declare that the research was conducted in the absence of any commercial or financial relationships that could be construed as a potential conflict of interest.

## Publisher’s Note

All claims expressed in this article are solely those of the authors and do not necessarily represent those of their affiliated organizations, or those of the publisher, the editors and the reviewers. Any product that may be evaluated in this article, or claim that may be made by its manufacturer, is not guaranteed or endorsed by the publisher.
