# Supplementary material for: Pre-Treatment Computed Tomography Radiomics for Predicting the Response to Neoadjuvant Chemoradiation in Locally Advanced Rectal Cancer: A Retrospective Study
Source: Front Oncol. 2022 May 10;12:850774. doi: 10.3389/fonc.2022.850774 (PMC9127861; doi:10.3389/fonc.2022.850774)
Supplement: Supplementary file 1 [file DataSheet_1.docx]

**Supplementary files:**

**Figure S1.** Radiomic feature selection by the least absolute shrinkage and selection operator (LASSO) model. **(A)** Penalty parameter (λ) selection in LASSO using 10-fold cross-validation. The area under the curve (AUC) of the receiver operating characteristic curve is plotted versus log (λ) with the base of constant Euler number *e*. The left and right dotted vertical lines represent the corresponding log (λ) values selected by the minimum criterion, and the one standard error of the minimum criterion (the 1-SE criterion), respectively. We chose the 1-SE criterion in the present study, in which log (λ) = -3.3894 and λ = 0.0337. **(B)** LASSO coefficient shrinkage plot of the radiomics features. The coefficient of each feature is plotted versus log (λ). The two vertical lines represent similar meanings as panel A, and the optimal log (λ) by the 1-SE criterion shows 11 nonzero coefficients.


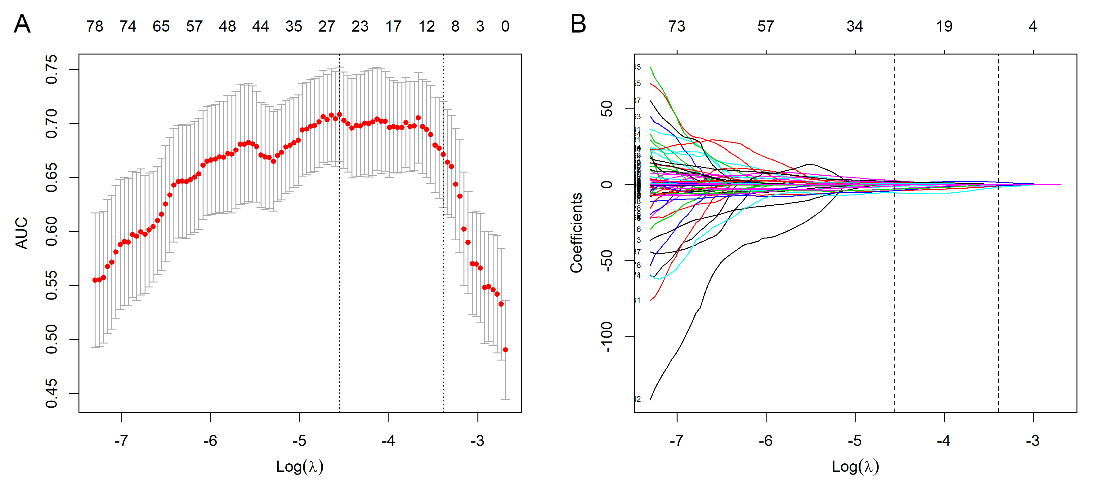


**Figure S2.** Calculation of Rad-score (the calculation was run in R software version 3.6.1)

Rad-score = dgr_GLevNonU45*-0.00226862648895624+dgr_RLNonUni45*-1.21266235549903e-05+S_0_5_Correlat*-0.880988834407511+S_4_0_Correlat*1.64337257203533+S_5_5_Correlat*-0.698798328567499+S_5_5_InvDfMom*-1.62601936637278+Skewness*0.312519639575789+Teta2*0.0216229062853876+Variance*0.000679311722035396+WavEnLH_s_3*-0.0211575015138424+WavEnLH_s_4*-0.000422699607692113 + 0.132361247698776

**Table S1. Assessment of the radiomics quality score (RQS) for the present study**

| **No.** | **Item** | **Item Score** | **Score for our study** |
| --- | --- | --- | --- |
| 1 | Image protocol quality - well-documented image protocols (for example, contrast, slice thickness, energy, etc.) and/or usage of public image protocols allow reproducibility/replicability | + 1 (if protocols are well-documented) + 1 (if public protocol is used) | 1 |
| 2 | Multiple segmentations - possible actions are: segmentation by different physicians/algorithms/software, perturbing segmentations by (random) noise, segmentation at different breathing cycles. Analyze feature robustness to segmentation variabilities | + 1 | 1 |
| 3 | Phantom study on all scanners - detect inter-scanner differences and vendor-dependent features. Analyze feature robustness to these sources of variability | + 1 | 0 |
| 4 | Imaging at multiple time points - collect images of individuals at additional time points. Analyze feature robustness to temporal variabilities (for example, organ movement, organ expansion/ shrinkage) | + 1 | 0 |
| 5 | Feature reduction or adjustment for multiple testing - decreases the risk of overfitting. Overfitting is inevitable if the number of features exceeds the number of samples. Consider feature robustness when selecting features | - 3 (if neither measure is implemented) + 3 (if either measure is implemented) | 3 |
| 6 | Multivariable analysis with non-radiomics features (for example, EGFR mutation) - is expected to provide a more holistic model. Permits correlating/inferencing between radiomics and non-radiomics features | + 1 | 1 |
| 7 | Detect and discuss biological correlates - demonstration of phenotypic differences (possibly associated with underlying gene–protein expression patterns) deepens understanding of radiomics and biology | + 1 | 1 |
| 8 | Cut-off analyses - determine risk groups by either the median, a previously published cut-off or report a continuous risk variable. Reduces the risk of reporting overly optimistic results | + 1 | 1 |
| 9 | Discrimination statistics - report discrimination statistics (for example, C‑statistic, ROC curve, AUC) and their statistical significance (for example, p‑values, confidence intervals). One can also apply resampling method (for example, bootstrapping, cross-validation) | + 1 (if a discrimination statistic and its statistical significance are reported) + 1 (if a resampling method technique is also applied) | 2 |
| 10 | Calibration statistics - report calibration statistics (for example, Calibration-in‑the-large/slope, calibration plots) and their statistical significance (for example, *P*‑values, confidence intervals). One can also apply resampling method (for example, bootstrapping, cross-validation) | + 1 (if a calibration statistic and its statistical significance are reported) + 1 (if a resampling method technique is also applied) | 2 |
| 11 | Prospective study registered in a trial database - provides the highest level of evidence supporting the clinical validity and usefulness of the radiomics biomarker | + 7 (for prospective validation of a radiomics signature in an appropriate trial) | 0 |
| 12 | Validation - the validation is performed without retraining and without adaptation of the cut-off value, provides crucial information with regard to credible clinical performance | - 5 (if validation is missing) + 2 (if validation is based on a dataset from the same institute) + 3 (if validation is based on a dataset from another institute) + 4 (if validation is based on two datasets from two distinct institutes) + 4 (if the study validates a previously published signature) + 5 (if validation is based on three or more datasets from distinct institutes)  *Datasets should be of comparable size and should have at least 10 events per model feature | 2 |
| 13 | Comparison to ‘gold standard’ - assess the extent to which the model agrees with/is superior to the current ‘gold standard’ method (for example, TNM-staging for survival prediction). This comparison shows the added value of radiomics | + 2 | 2 |
| 14 | Potential clinical utility - report on the current and potential application of the model in a clinical setting (for example, decision curve analysis). | + 2 | 2 |
| 15 | Cost-effectiveness analysis - report on the cost-effectiveness of the clinical application (for example, QALYs generated) | + 1 | 0 |
| 16 | Open science and data - make code and data publicly available. Open science facilitates knowledge transfer and reproducibility of the study | + 1 (if scans are open source) + 1 (if region of interest segmentations are open source) + 1 (if code is open source) + 1 (if radiomics features are calculated on a set of representative ROIs and the calculated features and representative ROIs are open source) | 4 |
| Total points (36 = 100%) | | | 22 |
